# Fibrin(ogen) Is Constitutively Expressed by Differentiated Intestinal Epithelial Cells and Mediates Wound Healing

**DOI:** 10.3389/fimmu.2022.916187

**Published:** 2022-06-22

**Authors:** Amira Seltana, Gabriel Cloutier, Vilcy Reyes Nicolas, Taoufik Khalfaoui, Inga C. Teller, Nathalie Perreault, Jean-François Beaulieu

**Affiliations:** Department of Immunology and Cell Biology, Faculty of Medicine and Health Sciences, Université de Sherbrooke, Sherbrooke, QC, Canada

**Keywords:** fibrinogen, epithelial restitution, intestinal homeostasis, human, Crohn’s disease, dabigatran, mouse, PI3K

## Abstract

Fibrinogen is a large molecule synthesized in the liver and released in the blood. Circulating levels of fibrinogen are upregulated after bleeding or clotting events and support wound healing. In the context of an injury, thrombin activation drives conversion of fibrinogen to fibrin. Fibrin deposition contains tissue damage, stops blood loss, and prevents microbial infection. In most circumstances, fibrin needs to be removed to allow the resolution of inflammation and tissue repair, whereas failure of this may lead to the development of various disorders. However, the contribution of fibrinogen to tissue inflammation and repair is likely to be context-dependent. In this study, the concept that fibrin needs to be removed to allow tissue repair and to reduce inflammation is challenged by our observations that, in the intestine, fibrinogen is constitutively produced by a subset of intestinal epithelial cells and deposited at the basement membrane as fibrin where it serves as a substrate for wound healing under physiological conditions such as epithelial shedding at the tip of the small intestinal villus and surface epithelium of the colon as well as under pathological conditions that require rapid epithelial repair. The functional integrity of the intestine is ensured by the constant renewal of its simple epithelium. Superficial denuding of the epithelial cell layer occurs regularly and is rapidly corrected by a process called restitution that can be influenced by various soluble and insoluble factors. Epithelial cell interaction with the extracellular matrix greatly influences the healing process by acting on cell morphology, adhesion, and migration. The functional contribution of a fibrin(ogen) matrix in the intestine was studied under physiological and pathological contexts. Our results (immunofluorescence, immunoelectron microscopy, and quantitative PCR) show that fibrin(ogen) is a novel component of the basement membrane associated with the differentiated epithelial cell population in both the small intestine and colon. Fibrin(ogen) alone is a weak ligand for epithelial cells and behaves as an anti-adhesive molecule in the presence of type I collagen. Furthermore, the presence of fibrin(ogen) significantly shortens the time required to achieve closure of wounded epithelial cell monolayers and co-cultures in a PI3K-dependent manner. In human specimens with Crohn’s disease, we observed a major accumulation of fibrin(ogen) throughout the tissue and at denuded sites. In mice in which fibrin formation was inhibited with dabigatran treatment, dextran sulfate sodium administration provoked a significant increase in the disease activity index and pathological features such as mucosal ulceration and crypt abscess formation. Taken together, these results suggest that fibrin(ogen) contributes to epithelial healing under both normal and pathological conditions.

## Introduction

The intestinal mucosa is part of the largest immune system of the body (1) consisting of a single layer of epithelial columnar cells that cover an underlying stromal tissue that containing immune, blood, and muscle cells. The epithelium provides a physical and functional selective barrier that allows absorption of nutrients, ions, and water while restraining the entry of luminal microorganisms and macromolecules. The efficiency of the epithelial barrier is ensured by cell–cell and cell–extracellular adhesion complexes, a continuous mucus layer and production of anti-microbial molecules (2, 3). Another key element for the maintenance of barrier efficiency is the rapid renewal of the epithelium, one of the fastest in the body, that relies on stem cells located at the base of the crypts for the production of progenitor cell populations that expand, differentiate, and up-migrate to replace cells lost by shedding at the tip of the villi in the small intestine and at the surface epithelium of the colon (4, 5). The mechanisms that regulate intestinal cell proliferation, migration, differentiation, and shedding are relatively complex but clearly involve interactions with the basement membrane, a specialized type of extracellular matrix composed of insoluble components originating from epithelial and subepithelial–stromal cells (6–8).

Intestinal epithelial cell–matrix interactions are also important in case of mucosal injuries occurring during normal gut functions and under pathologic circumstances (9–11). For instance, substantial alterations in the expression of various extracellular matrix molecules such as laminins and tenascins have been reported in Crohn’s disease (CD) (12, 13). Indeed, disrupted mucosal barrier integrity leads to excessive immune response to gut microbiota and luminal antigens that contribute to a proinflammatory environment that, in turn, alters epithelial homeostasis and thus contributes to the pathogenesis of inflammatory bowel diseases such as CD (14, 15).

Fibrinogen (FBG) is synthesized in hepatocytes and integrates the products of the three genes, namely, *FGA*, *FGB*, and *FGG*, which when assembled is secreted as a 340-kDa glycoprotein (16). Circulating levels of FBG are upregulated in the context of an acute phase response after bleeding or clotting events and to support wound healing (17). In the context of an injury, thrombin activation drives conversion of FBG to fibrin. Fibrin deposition through the action of thrombin and hemostasis contains tissue damage, stops loss of blood, and prevents microbial infection (18). In most circumstances, fibrin needs to be removed to allow inflammation resolution and tissue repair, whereas failure of removal may lead to the development of various disorders (17, 18). For instance, FBG-driven inflammation has been suggested to mediate primary tumor development in a mouse colon inflammation/cancer model (19). However, the contribution of FBG to tissue inflammation and repair is likely to be context-dependent (17, 18).

In this study, we report that, in the intestine, FBG is produced by a subset of intestinal epithelial cells and deposited at the basement membrane as fibrin where it serves as a substrate for wound healing under physiological conditions such as at sites of epithelial shedding at the tip of the small intestinal villus and surface epithelium of the colon as well as under pathological conditions that require rapid epithelial repair.

## Materials and Methods

### Human Intestinal Specimens

Intestinal specimens of healthy adults were obtained from Transplant Québec (Québec, Canada). Ileal specimens affected with CD and corresponding resection margins used for cryosectioning and mRNA extraction were obtained from therapeutic resections of consenting patients at the Centre Hospitalier Universitaire de Sherbrooke (CHUS), and diagnoses were confirmed by a pathologist as reported previously (12). Ileal samples from patients diagnosed with CD used for mRNA extraction were also obtained from the Cooperative Human Tissue Network (www.chtn.org). Colon cryosections were prepared from samples of the resection margins of consenting colorectal cancer patients treated at the CHUS as described previously (20). Mid-gestation (18–20 weeks) human small intestinal specimens were obtained from elective pregnancy terminations and were used for cryosectioning and mRNA extraction as well as the generation of primary cell cultures (21). For some experiments, mid-gestation ileum specimens were processed for the separation of epithelial and stromal fractions and mRNA extraction as previously described (22). The projects were in accordance with the protocols approved by the Institutional Human Research Review Board of the Université de Sherbrooke/CHUS.

### Animal Experiments and Tissues

All experiments were approved by the Animal Research Committee of the Faculty of Medicine and Health Sciences of the Université de Sherbrooke (approval number: FMSS-2017-2072) and followed the standards and policies of the Canadian Council on Animal Care in the Sciences. A minimum of four mice were used for each described condition.

#### Dextran Sulfate Sodium –Induced Colitis

For dextran sulfate sodium (DSS)–induced colitis in 60-day-old wild-type C57BL/6 male mice (Charles River, Sherbrooke, QC), 2%–5% (w/v) DSS [Molecular weight (MW): 35,000–50,000] (Chondrex, Woodinville, WA, lot: DB001-38) was dissolved in sterile drinking water provided *ad libitum* for 7 days as previously described (23). The severity of the disease was monitored by the evaluation of the disease activity index (DAI) each day according to the Cooper’s criteria (24) and scored as follows: loss of bodyweight, 0–3 (0 ≤ 1%, 1 = 1%–5%, 2 = 5%–10%, and 3 = 10%–20%); stool consistency, 0–4 (0, normal; 2, loose; and 4, diarrhea); and stool blood, 0–4 (0, normal; 2, visual or lightly colored; 4, and gross or heavily colored). At the end of these studies, mice were anesthetized with 2% isoflurane (VWR, Mississauga, ON) prior to being euthanized by carbon dioxide asphyxia. The colon and spleen were collected and monitored, and the overall DAI score was evaluated using a modified criteria from Cooper et al. (24), which include the criteria specified above plus rectal bleeding (0–4; 0, none; 2, moderate; and 4, heavy) and colon hardness (0–2; 0, normal; 1, mild; and 2, heavy). The overall DAI score represents the total of each of the four criteria divided by four.

#### Thrombin Inhibition

To investigate the fibrin effect on DSS-induced colitis, groups of mice were treated with the direct thrombin inhibitor Dabigatran Etexilate (Boehringer Ingelheim, Ingelheim am Rhein, Germany). The drug was administered orally at 10 mg/g of chow mixed with 25% (w/w) peanut butter to avoid the challenge associated with the taste of the drug. Mice were treated with dabigatran for 12 days including 5 days of pre-treatment following by 7 days of treatment with or without DSS. The effect of dabigatran on thrombin inhibition was estimated by the measurement of clotting time just before euthanasia. Under anesthesia, 1 ml of blood was taken by cardiac punction and deposited in a 37°C pre-warmed glass tube. The tube was shaken every 30 s until a clot formed.

#### Histopathology

Murine colons were fixed in 4% paraformaldehyde (Sigma-Aldrich, Oakville, ON) overnight at 4°C, dehydrated and embedded in paraffin. Tissue sections (5 µm) were cut and stained with hematoxylin and eosin as previously described (25). The tissue sections were scanned under a Nanozoomer 2.0-RS digital slide scanner (Hamamatsu Corporation, Bridgewater, NJ) to assess the histological grading of colitis. Epithelial damage, leucocyte infiltration, and histology were scored on the basis of the study by Dieleman et al. (26). Briefly, the extent of destruction of the mucosal architecture was scored 0–3 (0, normal; 1, mild; 2, moderate; and 3, extensive damage). The presence and degree of cellular infiltration was scored 0–3 (0, normal; 1, mild; 2, moderate; and 3, transmural infiltration). The extent of muscle thickening was scored 0–3 (0, normal; 1, mild; 2, moderate; and 3, extensive thickening). The presence or absence of goblet cells and crypt abscesses was scored 0–1 (0, absent; and 2, present). The histological score represents the sum of each criterion.

### Cell Culture

The human colon carcinoma cell line Caco-2/15 (27), a stable clone of the parental Caco-2 cell line [HBT37; American Type Culture Collection (ATCC), Rockville, MD], fully characterized elsewhere (28, 29), was grown under culture procedures detailed previously (30). The Caco-2/15 cell line was used for its ability to spontaneously initiate a differentiation program as an experimental cell model for villus epithelial cells (27–29, 31). The HIEC-6 cell line was generated from the mid-gestation fetal small intestine (32). HIEC-6 cells were grown according to (33) and used as an experimental epithelial cell model for intestinal crypt cells (31, 33). Primary cultures of human intestinal myofibroblast (HIM) cells were generated from the ileum of 16- to 18-week-old fetuses and kept in culture as described in previous studies (13). For the epithelial/stromal co-culture system, Caco-2/15 cells were seeded at high density onto a stromal HIM cell support, grown to confluence, and kept in culture for 14–20 days, as detailed previously (21, 34, 35). All cell cultures were routinely monitored for mycoplasma contamination. Caco-2/15 and HIEC-6 cell identities were confirmed by short-tandem repeat profiling cell authentication.

### Cell Binding Assays

Binding assays were carried out in flat-bottom 96-well tissue culture plates as described previously (36, 37), using a fibrinogen substrate as either a coating or a gel matrix. Well coatings were prepared using a fibrinogen solution (3 mg/ml) in the presence of thrombin (0.5 U/ml; Chemicon International) as described previously (38). Fibrin gel matrices were prepared according to the manufacturer’s instructions (Fibrin *In vitro* Angiogenesis Assay, ECM630, Chemicon International), whereas composite gels contained increasing percentages of fibrin: 10%, 20%, and 40% in type 1 collagen gel (2.5 mg/ml; BD Biosciences Pharmingen, Mississauga, ON). Coatings, fibrin matrices, and control wells were blocked with 2% bovine serum albumin (BSA) for 2 h at 37°C to reduce background binding. Caco-2/15 cells were harvested in 0.5 mM Ethylenediaminetetraacetic acid (EDTA) in 1× Phosphate-Buffered Saline (PBS), and 2 × 10^4^ cells were plated per well. Adhesion was pursued for 30 min for experiments carried out on coatings and overnight for experiments involving gel matrices. Unbound cells were removed by gently washing twice with 1× PBS, and the remaining cells were fixed in 4% paraformaldehyde/1× PBS for 1 h at 4°C, then permeabilized with 1% Triton X-100/1× PBS for 10 min. and stained with 4’,6’ diamidino-2-phenylindole (DAPI). The total number of DAPI-stained nuclei (bound cells) was expressed as a percentage of plated cells. These experiments were repeated at least three times, and a minimum of 400 cells were counted for each condition tested.

### Wound Assays

Wound-healing experiments were conducted as described in a previous study performed in our laboratory (35) under conditions that allow to specifically measure epithelial restitution (i.e., wound healing under a condition in which cell proliferation is inhibited). The proliferation-independent effect of epithelial restitution was ensured by treating all cultures with 2 mM hydroxyurea 48 h before and after wounding. Wounds were generated by superficial suction of either Caco-2/15 cells as a monolayer or on top of HIM in co-culture, immediately rinsed, and replenished with fresh complete media or complete media containing type I collagen (20 µg/ml; BD Biosciences Pharmingen, Mississauga, ON), human plasma fibronectin (20 µg/ml; Chemicon International), or fibrinogen (30 µg/ml; Chemicon International). All media contained hydroxyurea. Restitution of denuded cell areas was monitored over 48 h by phase-contrast microscopy, and the percentage of wound healing was established using MetaMorph cell imaging and analysis software (Universal Imaging Corporation, Downingtown, PA), as previously reported (35). In some experiments, Caco-2/15/HIM co-cultures were pre-treated 1 h with 15 µM of the Phosphoinositide 3-kinase (PI3K) inhibitor LY294002 in dimethylsulfoxide (DMSO) (39) before wounding and followed by medium replacement with fresh medium containing DMSO alone, LY294002 in DMSO, fibrinogen, or both LY294002 and fibrinogen for additional periods of 4 and 24 h before monitoring the restitution process.

### Indirect Immunofluorescence

Human and mouse intestinal tissues were embedded and prepared as previously described (20, 21, 36). Cells grown on coverslip and 3-µm cryo-tissue sections were fixed in 2% paraformaldehyde for 45 min, quenched in PBS-Glycine (200 mM, pH 7.4) for 45 min, and blocked in 5% non-fat powdered milk. Acetone fixation (10 min at −20°C) and 2% BSA blocking were also used with similar results. In some experiments, subconfuent Caco-2/15 cells were grown on coverslips and incubated in the presence of fibrinogen (3 mg/ml) for 24 h before fixation. Antibodies used in this work were mouse monoclonal anti-fibrinogen 15H12 (1/250; Abcam Inc., Cambridge, MA), Fluorescein Isothiocyanate (FITC)-conjugated polyclonal rabbit F0111 anti-fibrinogen (1/60; DakoCytomation, Mississauga, ON, Canada), mouse-monoclonal anti-fibrin 59D8 (40) (14 µg/ml; kind gift from Professor Charles T. Esmon, Oklahoma Medical Research Foundation), rabbit polyclonal anti-EHS laminin (1/500; Sigma-Aldrich, Oakville, ON), and FITC-conjugated α–smooth muscle actin (SMA) mouse monoclonal 1A4 (1/2000; Sigma-Aldrich). Secondary antibodies used were Alexa Fluor 488 or 590 anti-mouse or anti-rabbit antibodies (1/200; Invitrogen/ThermoFisher, Mississauga, ON). In some instances, when indicated, tissue sections or cell slides where counterstained with 0.01% Evan’s blue and/or DAPI, mounted with mounting medium (DAKO, High Wycombe, UK), and viewed with a DMRXA microscope (Leica) equipped for epifluorescence and digital imaging (MSP60 camera; Leica).

### Electron Microscopy Immunolocalization

Immunogold ultrastructural localization was carried out on human intestinal tissues at mid-gestation (18–20 weeks) as previously described (41). All steps involved in the preparation, fixation, and immunolocalization were carried out as detailed (42). Briefly, tissues were embedded in Lowicryl K4M resin at −20°C as previously described (43). Eighty-nanometer sections prepared on formvar-coated grids were incubated with the polyclonal rabbit F0111 anti-fibrinogen antibody diluted 1/50 in 1% BSA PBS (pH 7.4) and incubated overnight. A goat anti-rabbit secondary antibody conjugated to 10-nm gold particles was used for detection as previously described (42). Control staining was done by omitting the primary antibody. Images were taken with a HITACHI H7500 electron microscope.

### Intestinal Epithelium–Mesenchyme Dissociation and RT-PCR

Enriched fractions of epithelium and stroma were obtained from three mid-gestation human small intestines according to Perreault et al. (22). RNA was extracted from both fractions, and enrichment was confirmed by testing for tissue-specific markers by semi-quantitative RT-PCR. The primers were as previously reported for sucrase-isomaltase (44), tenascin-C (22), and RPLP0 (45). Primers used for the FBG β-chain were the forward primer: 5′-CTGTGGCCTACCAGGTGAAT-3′ and reverse primer: 5′-TGGTCCTGTTTTCTCCCATC-3′.

### Quantitative RT-PCR

Expression of FBG in HIEC-6 and Caco-2/15 cells as well as in control small intestinal samples was assessed by quantitative PCR (qPCR) using primers designed for the fibrinogen β-chain and RPLP0 as above. Assessment of gene expression was established according to the Pfaffl mathematical model (46) using RPLP0 as a validated reference gene for qPCR normalization (45). Expression of FBG was also evaluated in samples of mouse colon treated or not with dabigatran and DSS as described above using the mouse-specific primers for the fibrinogen β-chain (forward primer: 5′-TCAAACTCAAGCTGCCGATG-3′ and reverse primer: 5′-GTCAACAGGTCGATGACCAC-3′) as well as B2m, Rplp0, and Gusb as reference genes (47) using melting temperatures at 55°C, 59°C, 56°C, and 59°C, respectively. FBG expression was also tested in human small intestinal CD samples (active *vs.* resection margins) using the specific primers for human fibrinogen β-chain (forward primer: 5′-GCAACCAACACAGATGGGAA-3′ and reverse primer: 5′-CCATCCTGGTAAGCTGGCTA-3′) as well as RPLP0, B2M, and RPS3A as reference genes as published before (45, 48) using melting temperatures at 55°C, 57°C, 58°C, and 57°C, respectively. Real-time experiments were performed using an Mx3000P (Stratagene, La Jolla, CA) as previously described (45).

### Western Blot Analyses

Caco-2/15 cells were kept in culture, and protein extracts were collected on different days of confluence. Total protein (50 μg/ml) was separated on a 2% vertical agarose gel to detect the expression of native fibrinogen. Agarose gel electrophoresis was performed as previously described (49). For the preparation of cell-deposited extracellular matrix, post-confluent Caco-2/15 cells grown on plastic culture dishes were incubated in 27 µM ammonium hydroxide (NH_4_OH) solution as described previously (41). The remaining material was solubilized in 1× Laemmli buffer [2.3% sodium dodecyl sulfate (SDS), 10% glycerol, 0.005% bromophenol blue in 62.5 mM Tris-HCL, pH 6.8, containing 5% β-mercaptoethanol]. Proteins were separated on SDS-polyacrylamide gel electrophoresis (PAGE) 8% gels and electrotransferred onto nitrocellulose membranes (BioRad, Mississauga, ON). Membranes were blocked in 1× PBS containing 5% powdered skim milk and then incubated overnight with mouse anti-fibrinogen 15H12 antibody diluted 1/500 in the blocking solution. Immunoreactive bands were visualized using enhanced chemiluminescence (ECL) (GE Healthcare) according to the manufacturer’s instructions.

### Data Presentation and Statistical Analyses

All experiments were performed at least in triplicate. ANOVA using the Bonferroni’s multiple comparison test was used to analyze the results, and the Wilcoxon matched pairs signed-rank test was used when comparing pairs. Data were considered to be significantly relevant at *p* < 0.05 and are presented as mean ± SEM. Statistical calculations were performed using Prism 9.3 software (GraphPad Software, San Diego, CA).

## Results

### FBG Is Expressed by Human Intestinal Epithelial Cells

**Figure 1:**
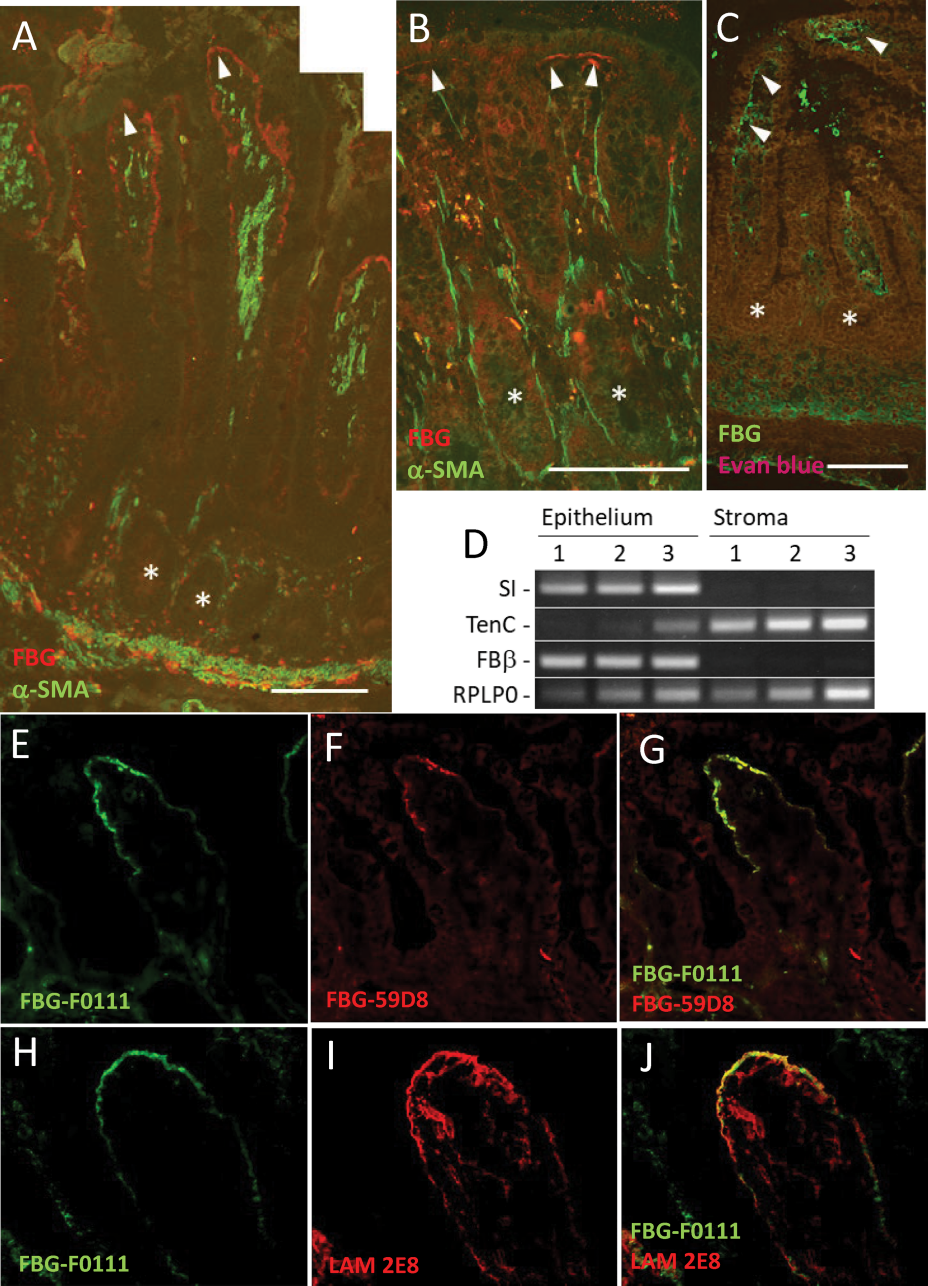
Epithelial FBG expression in the human intestinal mucosa. **(A, B)** Indirect immunofluorescence using the anti-FBG 15H12 antibody in red and α-SMA in green confirmed FBG deposition in the normal human intestine. Immunoreactive FBG was detected at the epithelial–stromal interface of the upper third regions of the villus in the adult small intestine **(A)** and at the base of the colonocytes of the surface epithelium of the adult colon **(B)**. FBG was also detected in the human fetal intestine at the epithelial–stromal interface in the upper half of the villi with the anti-FBG F0111 antibody in green (tissues were counterstained with Evan blue—brown-red staining). **(C)** No specific anti-FBG staining was detected in the region of the crypts (* in **A–C**). **(D)** Analysis of stromal and epithelium RNA fractions confirms the epithelial origin of FBG in the human gut. RT-PCR analysis of sucrase-isomaltase (SI), an exclusive intestinal epithelial cell marker, and tenascin-C (TenC), a stromal cell marker, confirmed that the FBG beta chain (FBβ) was expressed in the epithelial fraction of the mid-gestation intestine. RPLP0 was used as a loading control. **(E–G)** Representative double staining illustration of FBG at the tip of the villus using the anti-FBG F0111 antibody in green **(E, G)** and anti-fibrin 59D8 in red **(F,G)** showing that a significant part of FBG at this site is under the form of fibrin. **(H–J)** Representative double staining illustration for the immunodetection of FBG with the anti-F0111 antibody in green **(H, J)** and the anti-laminin beta chain 2E8 antibody in red **(I, J)** at the tip of the villus showing similar deposition at the epithelial–stromal interface. Scale bars: **(A, B)** 100 µm and **(C)** 50 µm.

Indirect immunofluorescence using anti-FBG antibodies was first performed to characterize the expression of FBG in the human intestine. In the adult ileum, staining of FBG was clearly visible and concentrated at the epithelial–stromal interface in the upper third of the villus (**Figure 1A**, red staining) and at the base of mature colonocytes in the surface epithelium of the adult colon (**Figure 1B**), whereas no FBG expression was observed in the base of epithelial cells of the glands (asterisks in **Figures 1A, B**). At mid-gestation, the human fetal small intestine is considered to be functionally similar to the adult (5, 50), and FBG was also detected at the epithelial–stromal interface of the upper half of the villus at this stage (**Figure 1C**, green staining). Because the main source of FBG is considered to be the liver (18), we investigated the intestinal origin and the tissue responsible for the FBG observed by RT-PCR using a procedure previously described for separating the intestinal epithelium from the underlying stroma (22). As shown in **Figure 1D**, the transcript encoding the β-chain was predominantly detected in the epithelial fractions of the mid-gestation small intestine, confirming its biosynthesis in the human intestinal epithelium. The FBG β-chain transcript expression in the intact adult small intestine was also confirmed by qPCR [Cycle threshold (Ct) values for FBG: 28.2 ± 1.4 vs. Ct RPLPO: 17.7 ± 1.4, mean ± SEM, n = 4].

Another intriguing question pertained to the form of FBG detected at the epithelial–stromal interface, considering that soluble FBG needs to be cleaved by thrombin to become insoluble as fibrin. Using an anti-FBG and anti-fibrin specific antibodies, double staining revealed that most of the FBG detected at the epithelial–stromal interface in the tip of the villus was in the form of fibrin (**Figures 1E–G**). Finally, because FBG appears to be deposited by epithelial cells, its contribution to the intestinal epithelial basement membrane was investigated. Double staining of FBG with laminin, a well-characterized basal lamina component of the intestinal epithelium, showed a similar staining pattern, suggesting that both components are located closely under epithelial cells at the base of the villus tip (**Figures 1H–J**).

**Figure 2:**
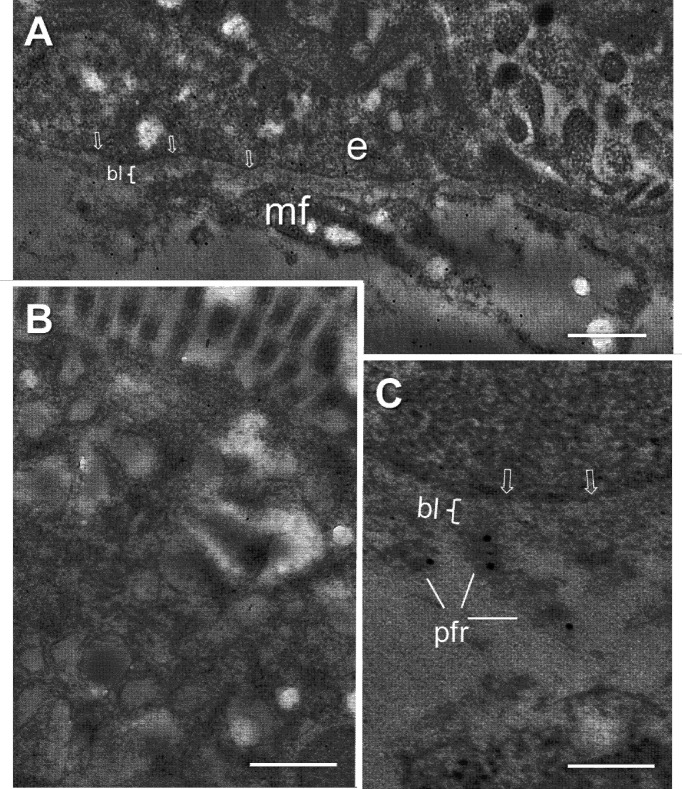
Ultrastructural immunolocalization of FBG at the intestinal epithelial–stromal interface. **(A)** Low magnification of the epithelial cell-stromal region showing positive intracellular staining at the base of the epithelial cells (e) and immunoreactive material over the extracellular components surrounding the subepithelial myofibroblasts (mf) with the exception of the epithelial basal lamina (bl) where only low-density gold staining is observed. **(B)** Low magnification of the apical region of an epithelial cell showing only background staining. **(C)** High magnification of the epithelial–stromal interface showing the lack of immunoreactive staining at the base of the epithelial cell, plasma membrane (arrows), and basal lamina (bl) while immunogold particles over the extracellular material of the pars fibroreticularis (pfr). Scales bars: **(A, B)** 500 nm and **(C)** 200 nm.

Immunoelectron microscopy was then used to investigate this question more specifically. FBG immunodetection was performed on ultrathin Lowicryl KM4 sections of mid-gestation small intestine. Immunogold particles were found in the cytoplasm at the base of the epithelial cells and in the underlying stromal region (**Figure 2A**), whereas only background labeling was detected in the apical part of the same cells (**Figure 2B**). The extracellular labeling was associated with various basement membrane structures underneath the epithelium and surrounding subepithelial myofibroblasts but appeared to be excluded from the basal lamina itself (**Figures 2A, C**).

### FBG Mediates Intestinal Epithelial Cell Wound Healing

**Figure 3:**
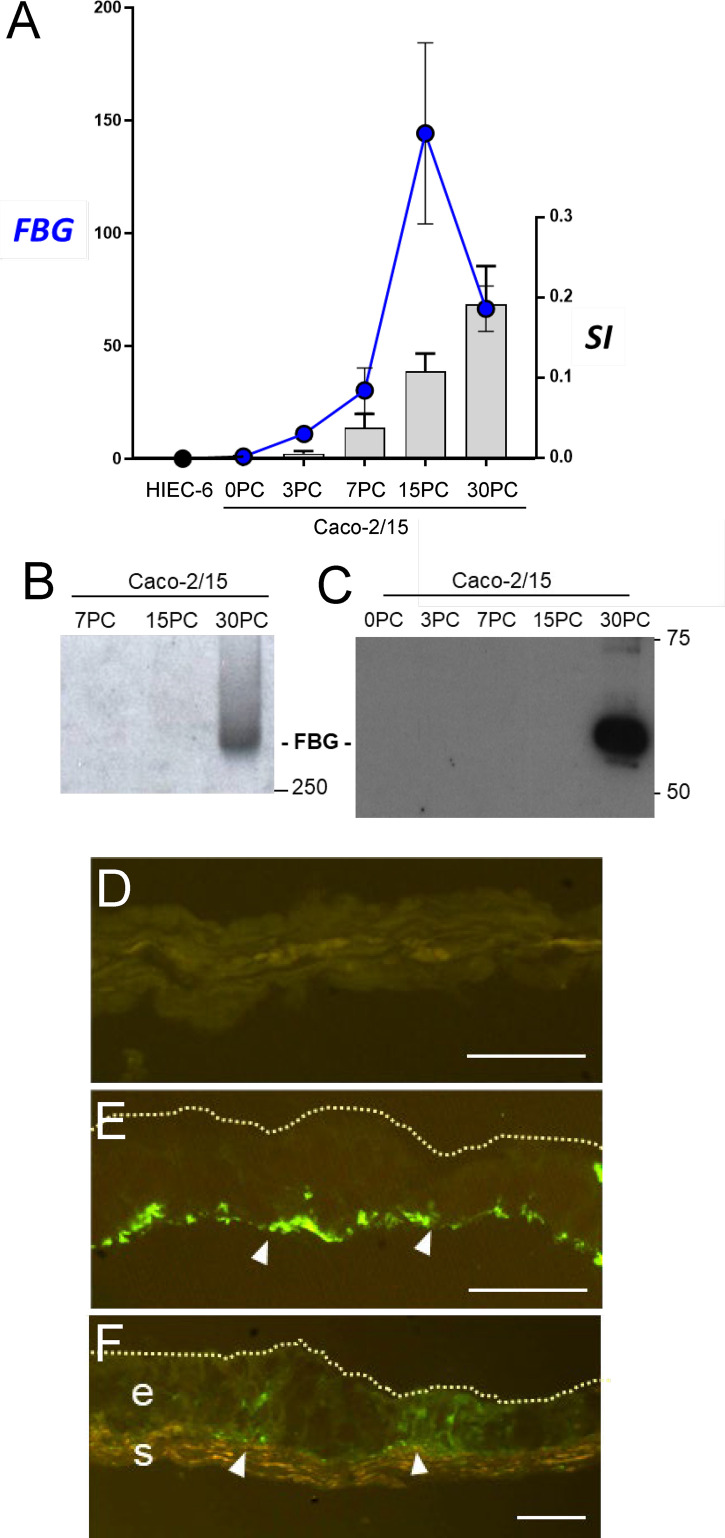
The spatial-temporal expression of FBG in intestine epithelial cells. **(A)** Expression of FBG in epithelial intestinal cell models that recapitulate the crypt-villus axis revealed an increase of expression of the FBG β-chain transcript in Caco-2/15 cells, closely accompanying the expression of the differentiation marker sucrase-isomaltase (SI) over the first 15 days of post-confluence (PC), whereas neither FBG nor SI was detected in HIEC-6 cells. Data are expressed as fold relative to small intestinal extracts. **(B, C)** When investigated at the protein level, FBG was only detected at later stages of post confluent Caco-2/15 cell culture either under its native 340-KDa form **(B)** or as insoluble extracellular material deposited by post confluent Caco-2/15 cells detected as 55kDa FBG fragment **(C)**. **(D–F)** Immunofluorescent detection of FBG with the anti-FBG F0111 antibody (in green) on cryosections of mono- and co-cultures cells. The FBG was found to accumulate at the base (arrowheads) of post confluent Caco-2/15 monolayers **(E)**. FBG was not detected in the multilayer of fetal stromal cells grown alone **(D)**. In co-cultures of Caco-2/15 cells (e) on top of fetal stromal (s) cells, FBG staining was observed at the base of the epithelial cells and at the epithelial–stromal interface (arrowheads) **(F)**. Scale bars: 25 µm.

The restricted expression pattern of FBG in differentiated intestinal epithelial cells was further characterized using well-established experimental cell models. qPCR analyses were performed on total RNA extracted from proliferative normal human intestinal epithelial crypt (HIEC) cells, a crypt cell model, and from Caco-2/15 cells, a villus enterocyte-like cell model, and expressed as fold relative to the adult small intestine (data provided above). As shown in **Figure 3A**, *FBG* β-chain mRNA transcripts were detected in late post-confluent Caco-2/15 cells. The levels of transcript expression gradually increased in parallel with sucrase-isomaltase (*SI*) expression, which is used as an indicator of intestinal cell differentiation (**Figure 3A**). *FBG* and *SI* mRNA were not detected in HIEC-6 cells (**Figure 3A**).

We next verified whether Caco-2/15 cells could, first, produce a mature FBG protein and, second, deposit FBG in the ECM of these cultures. Western blot analyses on whole cell lysates revealed that only fully differentiated Caco-2/15 cells at 30 days of post-confluence (PC) expressed detectable amounts of FBG under its mature native form at the expected molecular weight of 340 KDa (**Figure 3B**). The insoluble ECM matrix was isolated from Caco-2/15 cell cultures at different stages of confluence, and, under reduced conditions, the 60-kDa FBG form was detected from matrix extracts of fully differentiated 30-day PC Caco-2/15 cells (**Figure 3C**). FBG deposition was further evaluated by indirect immunofluorescence in post-confluent Caco-2/15 cell monolayers grown on plastic (**Figure 3E**) and in co-culture on intestinal myofibroblasts (**Figure 3F**), which revealed expression and basal deposition of immunoreactive FBG as compared to intestinal myofibroblasts alone used as control (**Figure 3D**).

**Figure 4:**
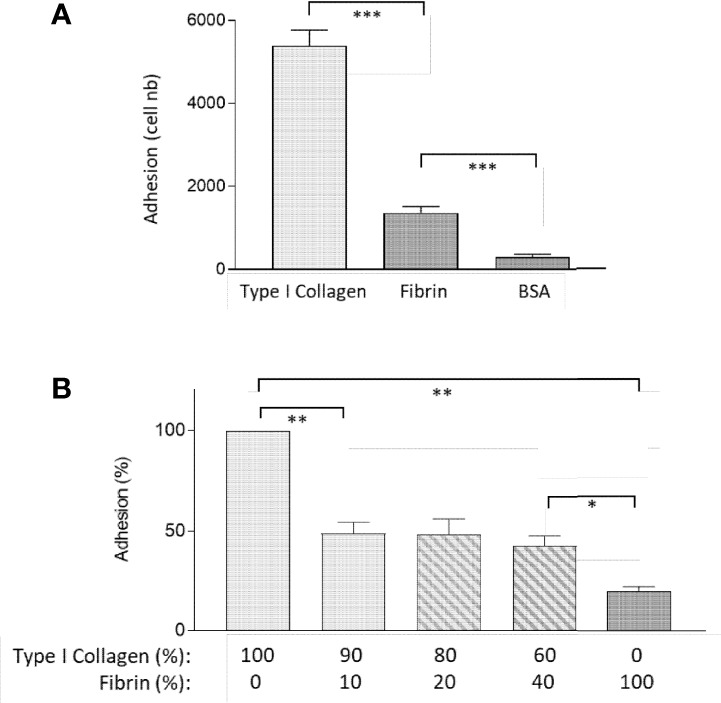
Fibrin acts as an anti-adhesive ECM for epithelial cells. **(A)** Caco-2/15 showed a 70% decrease of adhesion on fibrin coating as compared to type I collagen in 30-min adhesion assay. **(B)** Combined with collagen I, fibrin negatively affects the adhesive properties of the collagen gel, even when added at a low concentration, in an overnight adhesion assay. At least three independent experiments were performed for each condition, and the results are expressed as means ± SEM. *p < 0.05; **p < 0.01; ***p < 0.001.

We next sought to identify the function of FBG in intestinal epithelial cells by testing FBG as a cell adhesion substrate for Caco-2/15 cells. Cell adhesion assays on substrate coatings revealed that fibrin was a weak ligand for cell binding, as compared to type I collagen, a standard ECM substrate (**Figure 4A**). Comparative adhesive abilities were noted when both type I collagen and fibrin were prepared as pure gels (**Figure 4B**). Unexpectedly, the addition of fibrin to collagen gel preparations even in low proportions resulted in a significant drop of cell adhesion as compared to a pure collagen gel (**Figure 4B**). This was true for each proportion tested; most notably, the presence of only 10% fibrin was sufficient to interfere with binding to type I collagen to the same extent as higher percentages of 20% and 40%. These results suggested that fibrin exerted anti-adhesive properties.

**Figure 5:**
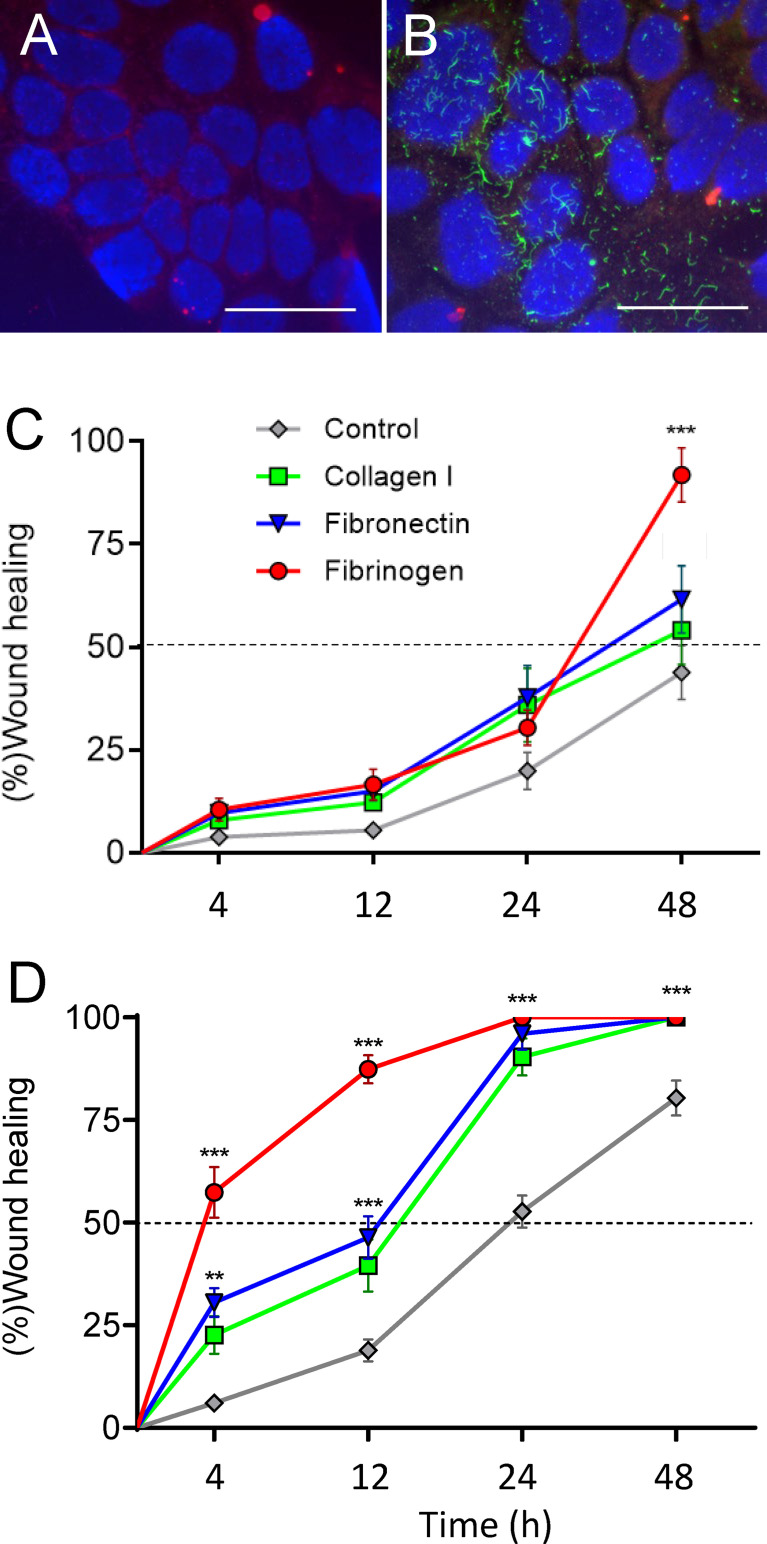
Assembly of FBG by intestinal epithelial cells and promotion of epithelial restitution on plastic and co-culture. **(A, B)** Caco-2/15 cells were grown on glass coverslips and then overlaid with medium alone **(A)** or containing plasma FBG **(B)** for 24 h before washing and processed for immunodetection of FBG and stained with DAPI. In comparison with control **(A)**, cells incubated with FBG showed a fibrillar pattern of deposition at their ECM **(B)**. Scale bars: 50 µm. **(C,D)** Caco-2/15 cells were grown to 12 days PC before micro-wounded and treated with hydroxyurea to evaluate the effect of extracellular components added to the medium on epithelial restitution. On plastic **(C)**, cell migration was not accelerated by the addition of type 1 collagen or fibronectin, whereas FBG had an effect after 48 h. In co-cultures with stromal cells **(B)**, all treatments stimulated cell migration compared to plastic assays so that wound healing was complete after 24 h for the three tested components. FBG was almost twice as efficient as collagen I or fibronectin so that 50% of the wound closure was reached in less than 4 h and more than 85% 12 h after wounding. At least three independent experiments were performed, and the results are expressed as means ± SEM. **p < 0.01; ***p < 0.001 vs. control at the same time.

The endogenous production of FBG by the intestine combined with the ability of FBG to act as a ligand for intestinal epithelial cells suggests that this protein could be involved in, or could contribute to, wound healing in the intestinal epithelium. As shown previously for fibronectin, intestinal cells have the ability to integrate soluble ECM components into their own ECM (51, 52). To determine whether intestinal epithelial cells could also incorporate exogenously added FBG into the ECM, complete plasma FBG was added to the culture medium of Caco-2/15 cells at 80% confluency for a 24-h period. Cells were then stained for FBG by indirect immunofluorescence and results showed the formation of a fibrillar pattern at their basal cell surface (**Figures 5A, B**). These results indicated that intestinal epithelial Caco-2/15 cells not only are capable of correctly synthesizing and processing FBG but also can assemble exogenous FBG into a fibrillar pattern.

The effect of FBG on intestinal epithelial restitution was then assessed first using Caco-2/15 cells grown on plastic to day 12 PC and micro-wounded (500–600 µm diameter) at multiple sites as described previously (35). The rate of wound closure was monitored in the presence of soluble type I collagen, fibronectin, or FBG added directly to the media and compared to complete media alone. In control conditions, approximately 50% of the wound area had been covered by 48 h (**Figure 5C**). In the presence of type I collagen or fibronectin, the rate of wound closure of epithelial cell monolayers grown on plastic was unaffected but the presence of FBG stimulated epithelial restitution to approximately 90% of the wound area after 48 h (**Figure 5C**). Epithelial restitution was previously shown to be more efficient in Caco-2/15-HIM co-cultures (35), which form a basement membrane–like structure containing most of the major protein components found in the epithelial basement membrane of the human small intestine (34, 35). Overall, type I collagen, fibronectin, and FBG significantly stimulated restitution after 24 h as compared to control (**Figure 5D**). However, FBG induced over 50% of wound closure after only 4 h and more than 85% after 12 h. These experiments underscore the striking stimulatory effect of FBG on intestinal epithelial restitution, which was further potentiated in the presence of a pre-existing basement membrane–like structure as shown with co-culture experiments.

**Figure 6:**
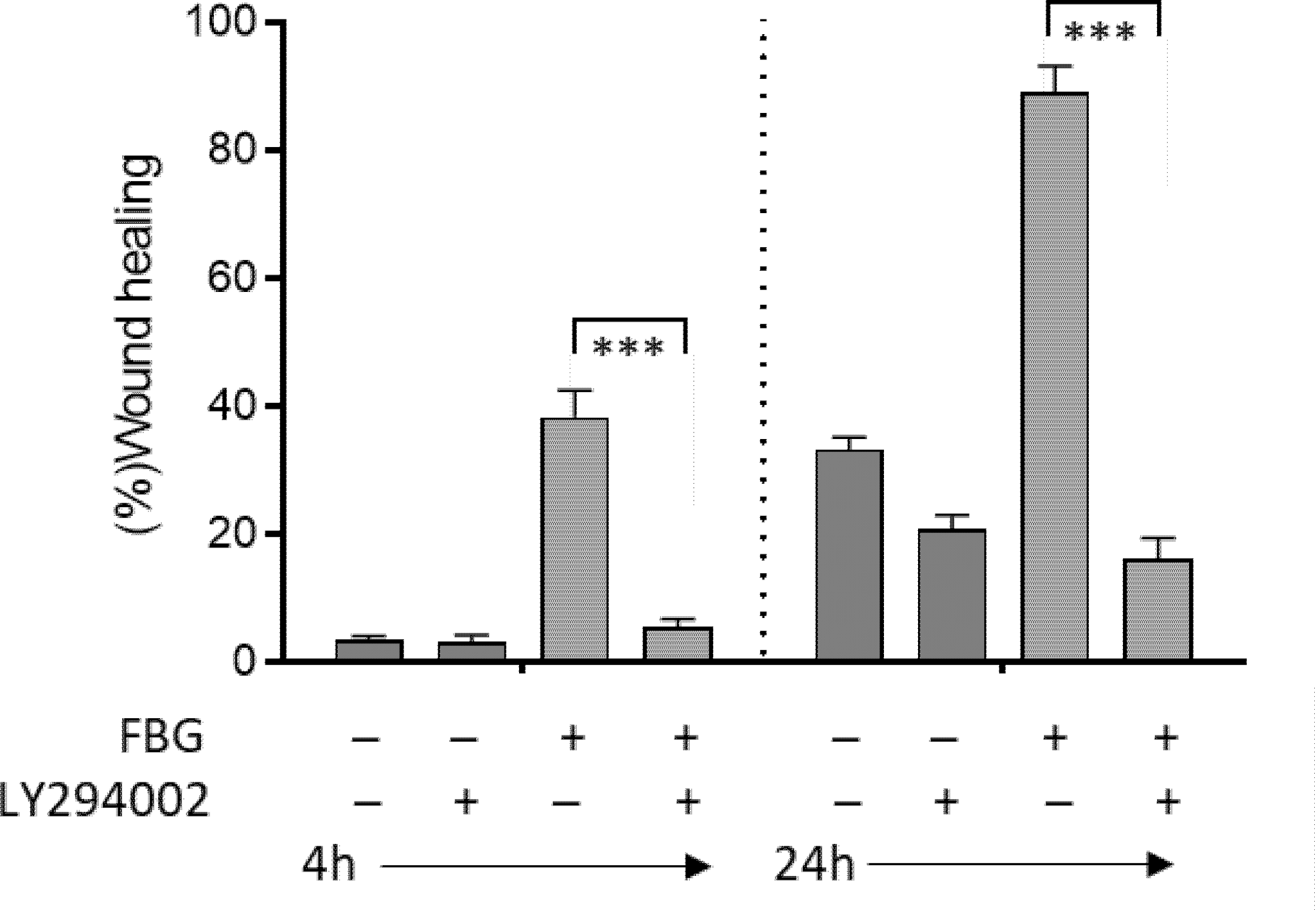
FBG migration cells were in a PI3K-dependent manner. Micro-lesions were induced in 12-day PC Caco-2/15 monolayers sitting on stromal cells, followed by PI3K inhibitor (LY294002) and/or FBG treatment for 4 or 24 h. LY294002 had no significant effect on Caco-2/15 grown without FBG. In cells grown in the presence of FBG, epithelial cell restitution was inhibited by LY294002 at both 4 and 24 h. At least three independent experiments were performed, and the results are expressed as means ± SEM. ***p < 0.001.

PI3K has been shown to regulate a major intracellular signaling pathway involved in the regulation of cell restitution (39, 53). To confirm that FBG promotes cell restitution *via* this canonical signaling pathway, wounded Caco-2/15-HIM co-cultures were incubated for 4 and 24 h with fresh medium supplemented or not with FBG and/or the PI3K inhibitor LY294002 vs. DMSO as a control. Inhibition of PI3K completely blocked FBG stimulated epithelial cell migration and reduced wound healing rates to the control culture level, indicating that FBG promotes epithelial cell restitution in a PI3K-dependent manner (**Figure 6**).

### FBG Is Increased in Specimens of Crohn’s Disease Patients

**Figure 7:**
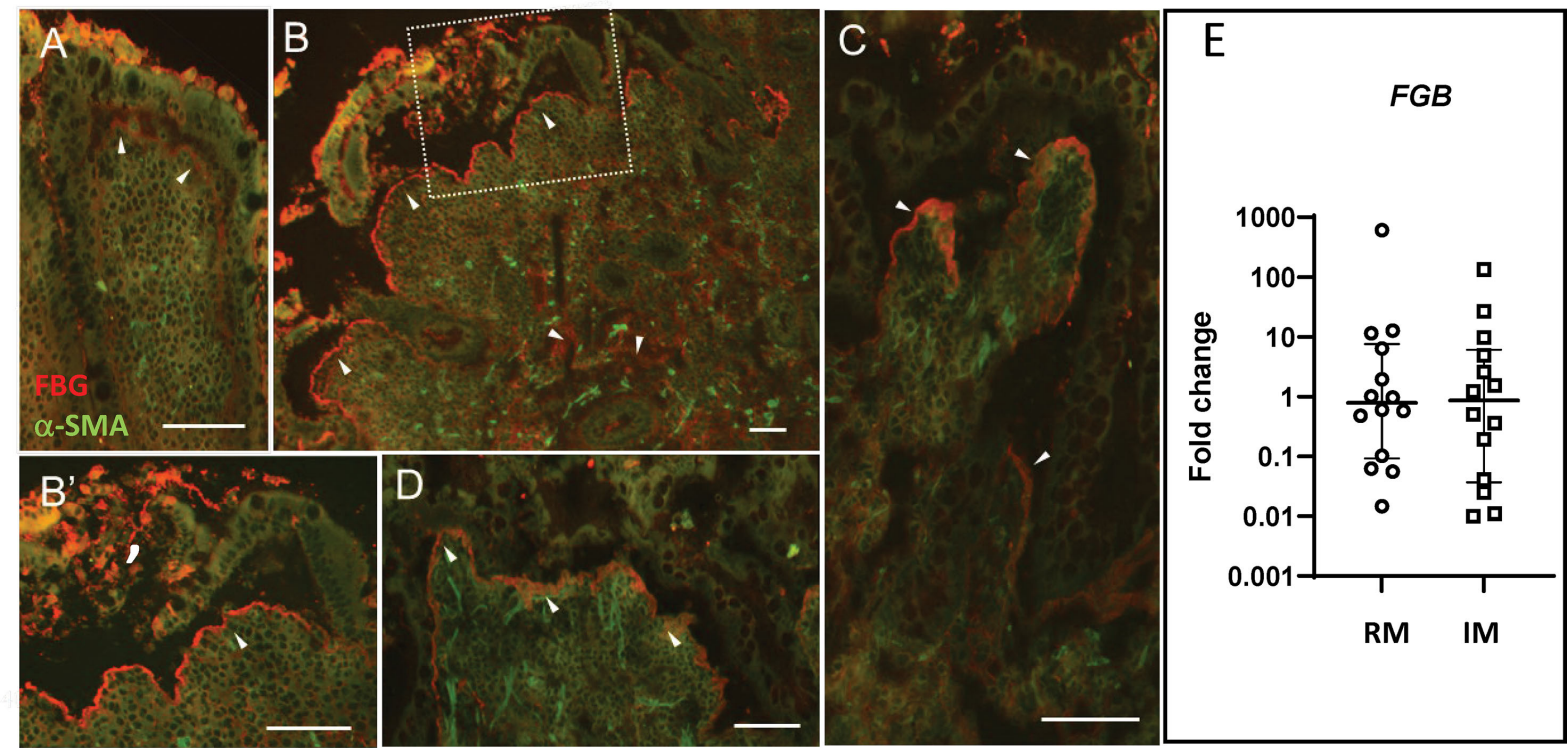
FBG expression in Crohn’s disease. Specimens from five patients with Crohn’s disease were tested for FBG expression using the anti-FBG F0111 antibody showed in red. **(A)** Resection margin showing FBG deposition at the epithelial–stromal interface (arrowheads). **(B)** Representative low magnification of inflamed mucosa showing more intense staining of immunoreactive FBG at the epithelial–stromal interface compared to resection margin. Note that staining at the epithelial–stromal interface was stronger in the inflamed mucosa than in the resection margin at sites where the epithelium was present **(C, D)** or where it was denuded **(B’)**. Additional staining was also detected throughout the lamina propria and blood vessels of the inflamed mucosa. Scale bars = 50 µm. **(E)** Scatter dot plot for *FBG* transcript analysis by qPCR in matched resection margin (RM) and inflamed mucosa (IM) paired samples from 14 patients with CD. Horizontal bars are for the medians with interquartile ranges.

Although epithelial cell restitution is a process occurring at the tip of the villus in the context of normal epithelial renewal (5), there are pathological situations where the intestinal epithelium is damaged, and a more active process of healing is required. CD is one of these types of pathologies (54). FBG expression was thus investigated by indirect immunofluorescence on specimens of small intestine affected with active CD. As shown in **Figure 7**, strong staining of immunoreactive FBG was observed in CD specimens (**Figures 7B–E**) as compared to control tissues from the resection margin (**Figure 7A**). FBG was noticeably concentrated at the epithelial–stromal interface at sites where the epithelium was present and in areas where it had been denuded (**Figures 7B–D**). Extensive staining was also detected throughout the lamina propria. qPCR analyses carried out on mRNA extracted from active CD specimens, and corresponding resection margins showed no significant difference (**Figure 7E**) in *FBG*, indicating that the apparent increase in the FBG deposition as detected *in situ* was not resulted from an upregulated *de novo* synthesis from the epithelium.

### FBG Deposition and Prevention of Intestinal Epithelial Damage

**Figure 8:**
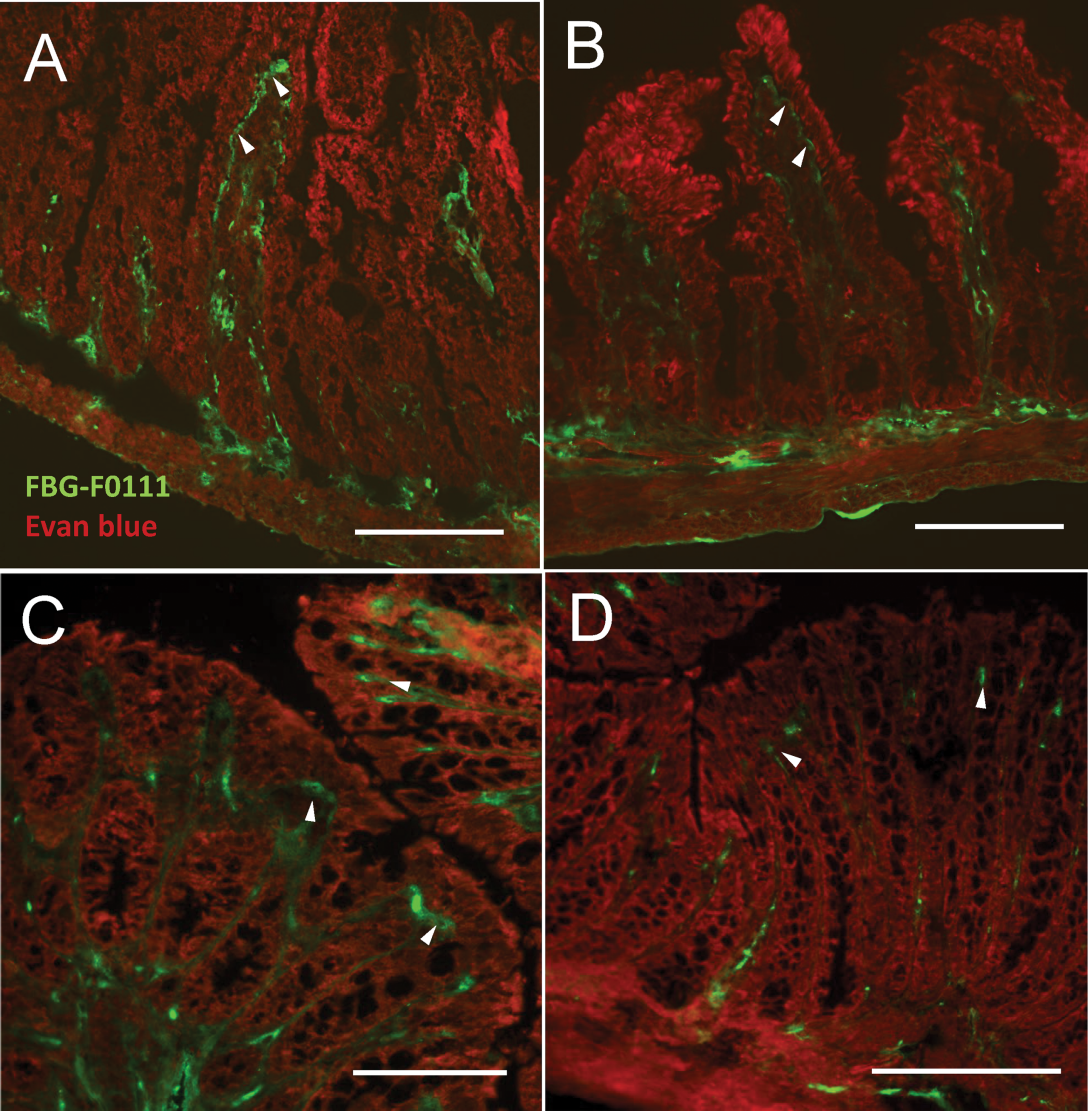
Effect of thrombin inhibitor dabigatran treatment on FBG deposition in the mouse intestine and blood coagulation. Mice were treated with dabigatran for 12 days and small intestine **(A, B)** and colon **(C, D)** were harvested for control **(A, C)** and treated **(B, D)**. Typical deposition of FBG (using the anti-FBG F0111) at the epithelial–stromal interface of the upper half of the villus and surface epithelium (green staining, arrowheads) as seen in the human intestine was observed in both the control mouse small intestine **(A)** and colon **(C)**. Consistent reduction of staining intensity in dabigatran treated mice was noted for both small intestine **(B)** and colon **(D)** compared to controls. Tissues were counterstained with Evan blue (red staining). Bars = 100 µm.

To further explore the role of FBG deposited at the epithelial–stromal interface in the promotion of epithelial wound healing under pathological conditions, we chose an animal experiment approach that would not interfere with overall FBG production but only impair FBG deposition under its insoluble fibrin form by inhibiting thrombin activity with dabigatran, a competitive thrombin inhibitor used in the clinic for attenuating thrombin activity and reducing fibrin formation for the prevention of stroke and various types of embolisms (55). Efficiency of the 12-day treatment was assessed by indirect immunofluorescence for the detection of immunoreactive FBG on cryosections of small intestine and colon and blood coagulation. As observed in the human ileum and colon, FBG was predominantly detected at the epithelial–stromal interface in the upper part of the villi in the small intestine (**Figure 8A**, green staining) and at base of the surface epithelium in the colon (**Figure 8C**) of control mice. Strong staining in blood vessels was also noted (**Figures 8A, B**). Immunoreactive FBG was also detected in tissues from dabigatran-treated mice for both the small intestine (**Figure 8B**) and colon (**Figure 8D**) but the intense staining seen in control was not observed. The loss of fibrin could not be confirmed by immunofluorescence with the mouse anti-fibrin antibody used on human tissue sections, but the significant increase in blood coagulation time (**Supplementary Figure 1**) indicated that the treatment was efficient although no other effect on the mice was noted.

We then used the DSS-induced colitis model (56) to evaluate the role of intestinal fibrin under conditions that challenge the integrity of the colonic epithelium. Before performing experiments combining dabigatran and DSS, we had to establish DSS treatment parameters. Initial experiments with C57BL/6 male mice were optimized with 2% DSS in drinking water as suggested (56). However, the efficiency of a new lot acquired for this set of experiments was found to be much less effective as evaluated by the change in body weight relative to control mice over a 7-day period with DSS 2% (102% ± 2.4; mean ± SEM, n = 4), 2.5% (103% ± 1.5), or 3% (97% ± 0.9). At 5% DSS, the average weight decreased to 91% ± 1.0 relative to controls (P < 0.03, Mann–Whitney test). We thus used the 5% concentration of DSS in drinking water for the next experiments.

**Figure 9:**
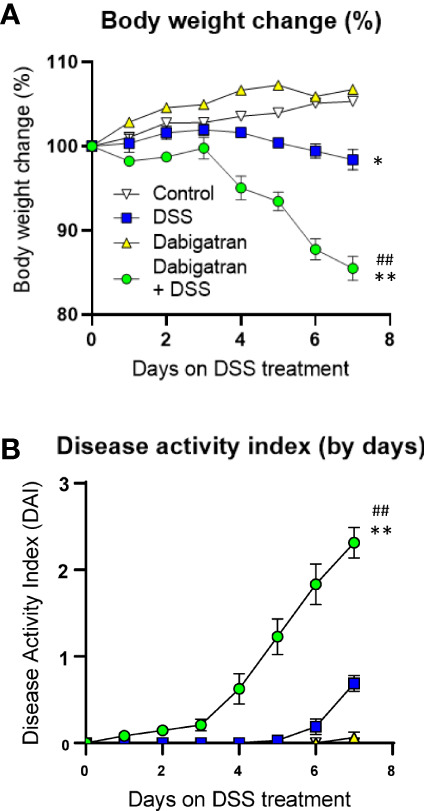
Thrombin inhibition exacerbated DSS-induced colitis in the mouse model. Mice treated or not with dabigatran for a 12-day period received DSS in their drinking water (or regular drinking water) for the last 7 days of treatment and the effects on body weight change **(A)** and disease activity index **(B)** were monitored daily showing that thrombin inhibition predisposes mice to DSS treatment with a significant reduction of weight and increase in disease activity index. *p < 0.05 and **p < 0.01, dabigatran/DSS vs. control; ^##^ *p* < 0.01, dabigatran/DSS vs. DSS alone.

Dabigatran or vehicle only was administrated 5 days before starting the 7 days of DSS treatments. DSS-induced colitis was followed by monitoring daily body weight loss (relative to day 0) and DAI scores. In contrast to control mice and those treated with dabigatran alone, mice receiving DSS showed a slight but statistically significant reduction of body weight in the last days of treatment, whereas the reduction for mice pre-treated with dabigatran that also received DSS was significantly more important (**Figure 9A**). The DAI score significantly rose under the same conditions (**Figure 9B**).

**Figure 10:**
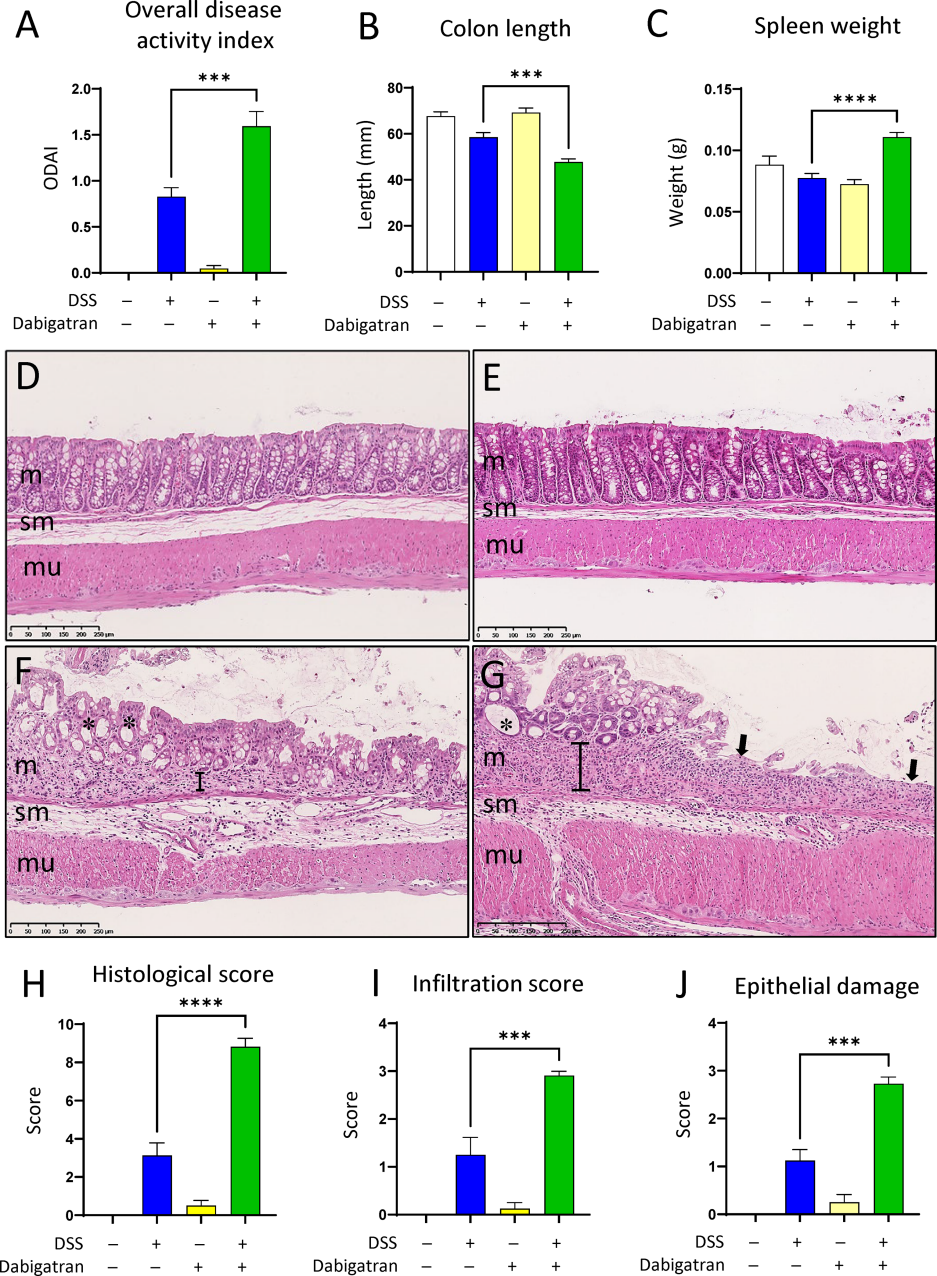
Thrombin inhibition worsens parameters associated with DSS-induced colitis. Mice treated or not with dabigatran for a 12-day period received DSS in their drinking water (or regular drinking water) for the last 7 days of treatment before euthanasia. In comparison to DSS alone, overall disease activity (feces consistence, fecal blood, rectal blood, and colon hardness) was found to be increased **(A)**, whereas colon length was further reduced **(B)** and a significant increase in spleen weight was noted **(C)**. Histological analysis of colon specimens showed that, in comparison with control **(D)** or dabigatran-treated **(E)**, the mucosa (m) of the DSS treated mice was altered with a loss of crypt architecture and leucocyte infiltration **(F)**, and these alterations were even more important in the dabigatran/DSS treated mice **(G).** In **(D–G)** m, mucosa; sm, submucosa; mu, muscle layers; *, crypt erosion; arrows, denuded epithelial regions; brackets, thickening of the underlying crypt stromal region. More important alteration in the intestines from dabigatran/DSS treated mice vs. DSS alone was confirmed by higher histological **(H)**, infiltration **(I)**, and epithelial damage **(J)** scores. n = 8–12, ***p < 0.001; ****p < 0.0001.

Severity of the colitis was also assessed by the overall DAI after euthanasia (**Figure 10**). DSS alone had a significant effect on the overall DAI, and although the dabigatran pre-treatment alone has no impact, it significantly exacerbated the experimental colitis when administered in combination with DSS (**Figure 10A**). A reduction in colon length (**Figure 10B**) and an increase in the spleen weight (**Figure 10C**) were also observed. Various alterations in the histological architecture of the colonic mucosa were noted for DSS and dabigatran/DSS treatments in comparison to controls and dabigatran alone including a loss a mucosal integrity, increase of leucocyte infiltration, and crypt abscesses (**Figures 10D–G**). The histology score was significant higher in the colon of mice treated with dabigatran/DSS than DSS alone (**Figure 10H**). Similar data were obtained for the infiltration score (**Figure 10I**) and epithelial damage (**Figure 10J**). Finally, when analyzed at the transcript level in the colon specimens, no significant difference in *FBG* levels was observed between the treatments (**Supplementary Figure 2**) although FBG protein accumulation into the mucosa of DSS-treated mice was noted by immunofluorescence as reported previously (57).

## Discussion

In this study, we report an endogenous production of fibrinogen in the intestinal mucosa and the deposition of fibrin as a constitutive basement membrane component at the epithelial–stromal interface of the villus tip of the small intestine and surface epithelium of the colon. *In cellulo* experimentation confirmed the FBG production by human intestinal epithelial cells and identified a direct role for this protein in the wound healing process. The accumulation of immunoreactive FBG in intestinal tissue affected by CD suggested a potential role for this anti-adhesive molecule in intestinal distress. Using the DSS mouse model, we showed that the inhibition of fibrin formation worsened DSS-induced colitis symptoms, indicating that epithelial fibrin(ogen) deposition is protective for mucosa integrity.

Up to now, extrahepatic sites of fibrinogen synthesis have been proposed for only a few normal tissues such as the lung or the brain, based on the detection of gamma chain FBG transcripts (58). FBG has been reported to be expressed in various non-hepatic carcinoma cell lines derived from the lung (59), uterus (60), breast (61), and colon (62). Interestingly, FBG expression in these cell models was relatively weak or incomplete under basal conditions but modulated by proinflammatory cytokines, suggesting that it may reflect an *in vivo* process associated with inflammation and repair (59, 62). The epithelial origin of FBG as confirmed at the transcript level in the intestine and its deposition at the tip of the villus and surface epithelium of the small intestine and colon, respectively, which are sites of the cell shedding process in these rapidly renewing epithelia (5), is consistent with this repair function. Such a role for FBG is not without precedent because, tenascin-C, another anti-adhesive extracellular matrix molecule also expressed according to a predominant gradient toward the tip of the intestinal villus and surface epithelium of the colon has been suggested to participate in the shedding process (63, 64).

The next question that we addressed about FBG at the intestinal epithelial–stromal interface was its type of fibrillar assembly. In blood clots, FBG is converted to fibrin through the proteolytic activity of thrombin, but, in the lung epithelial cell model, incorporation of FBG into the extracellular matrix was found to be independent of thrombin cleavage (65). However, intestinal epithelial cells are a significant source of thrombin (66), implying that the FBG produced by these cells is likely converted into fibrin. Immunofluorescence analysis using a fibrin-specific antibody (40) indicated that FBG accumulates at the intestinal epithelial–stromal interface under the form of fibrin. Previous studies reported that FBG was subject to a polarized secretion toward the basement membrane in the lung (59), but its precise location was not investigated. On the basis of indirect immunofluorescence double staining for immunoreactive FBG and laminin, a basal lamina specific component, FBG, appeared to be associated with the basement membrane, but ultrastructural immunolocalization showed that FBG was predominantly distributed with extracellular material that bridges the basal lamina and interstitial matrix components referred to as the pars fibroreticularis (67). The impact of this finding is difficult to evaluate at this time. One may speculate that being molecularly associated with the interstitial extracellular matrix, FBG-derived matrix, may remain available and functional for the sealing and wound healing in denuded regions of the epithelium.

To investigate the potential function of FBG in the intestinal epithelium, we used two cell lines that recapitulate the crypt-villus axis of the small intestine: HIEC-6 and Caco-2/15 cells (27–29, 31, 32). Interestingly, the original Caco-2 cells were reported to express basal levels of FBG (62), making them an attractive cell model for our work. FBG transcript expression in intestinal cells was directly related to cell differentiation as evaluated with the enterocyte marker sucrase-isomaltase, being not expressed in HIEC-6 and accumulating with confluence in Caco-2/15 cells. It is noteworthy that the quantitation of FBG expression was relative to intestinal extracts, indicating that Caco-2/15 cells expressed relatively high levels of FBG mRNA although the complete form of FBG protein was not detected in the first 15 days of post-confluent culture. Defective FBG assembly has been reported in breast carcinoma cells (61), and it is possible that a similar phenomenon occurs transiently in Caco-2/15 cells before 30 days of culture. FBG was also found to be deposited as insoluble material in the extracellular matrix of post-confluent Caco-2/15 cells maintained as monolayers on plastic as well as when grown at the surface of stromal cells, consistent with the expression of active thrombin by these cells (66). Another interesting characteristic of Caco-2/15 cells relative to FBG matrix assembly is their ability to incorporate exogeneous FBG components into an insoluble fibrillar matrix even at subconfluent stages when endogenous FBG is not produced.

The next question that was investigated relative to intestinal epithelial cell interaction with FBG was adhesiveness. As compared to type I collagen that allows a maximum of cell binding, only 20% of Caco-2/15 cells attached to fibrin under the same conditions, suggesting that it acts as a weak adhesive molecule. Cell adhesion to extracellular material can be mediated by a variety of receptors including integrins and intestinal cells express a large repertoire of them (68, 69). However, integrin receptors characterized to interact with FBG including the leucocyte integrin receptor αMβ2 and other members of the β2 subfamily (70, 71), integrin α5β1 (72), and integrin αVβ3 (73) are either not expressed or expressed at low levels in Caco-2/15 cells, consistent with its limited adhesiveness observed on this substrate. In contrast, keratinocytes that are completely devoid of these receptors fail to interact with fibrin(ogen) in the process of wound repair (74), suggesting that minimal adhesiveness may be required for efficient migration. In this context, it is interesting to note that inclusion of fibrin into the fibrillar collagen matrix even at low concentrations significantly reduced Caco-2/15 cell adhesion, suggesting anti-adhesion properties of fibrin and potentially mimicking the substrate composition that remains after epithelial denuding at the sites of intestinal cell shedding. Orend and Chiquet-Ehrismann (75) used the term adhesion-modulating protein to classify the inhibitory effect of tenascin-C on cell adhesion to fibronectin, but, in this case, cell signaling pathways controlling cell migration were supressed (76). The migratory potential of FBG on intestinal epithelial cells was thus investigated by evaluating the restitution potential of micro-wounded Caco-2/15 cells. On cells grown as monolayers on plastic, epithelial migration was only moderately stimulated by FBG as compared to other conditions. However, as shown previously, epithelial restitution was significantly accelerated for Caco-2/15 cells in a co-culture system with myofibroblasts (35, 77) and further enhanced by FBG that was even more efficient than collagen I and fibronectin. Finally, PI3K inhibition studies confirmed that FBG-enhanced cell restitution was acting to activate this intracellular signaling pathway involved in the regulation of cell restitution (39, 53). Taken together, these results indicate that FBG is deposited at the base of intestinal epithelial cells at sites of epithelial shedding where it can actively promote restitution.

We then investigated the potential role of FBG in pathologies involving intestinal epithelial destruction such as in CD, thus requiring efficient epithelial healing to prevent microorganism colonization (54). As expected, deposition of FBG was observed at the epithelial–stromal interfaces and at the remaining stroma of denuded epithelial regions in inflamed specimen of patients with CD. However, overall FBG expression in inflamed specimens was not found to be different to that determined from the resection margins as evaluated by qPCR. This observation is unexpected because FBG expression is modulated upon an inflammation-driven acute phase response (17), whereas several proinflammatory cytokines are overexpressed in the intestinal mucosa of CD (78). Pro-inflammatory cytokines such as interleukin-6 were shown to promote FBG expression in Caco-2 cells at both the protein (62) and transcript levels (Seltana A, unpublished). Because FBG is exclusively synthesized by intestinal epithelial cells, one may speculate that the lack of increase of FBG transcripts in the CD mucosa is the result of damaged epithelium. Extra-FBG accumulation in the CD mucosa as suggested by immunodetection would thus originate from the blood circulation (79), as suggested for other types of tissue injuries (18). To further investigate the possible role of FBG and fibrin at the epithelial–stromal interface under experimental conditions that challenge the integrity of the intestinal epithelium, we used the well-characterized DSS-induced colitis mouse model (56, 80). Considering that FBG is deposited at the epithelial basement membrane under a pro-migratory fibrin form as shown herein, which likely results from the co-expression of the required machinery for producing active thrombin (66), we chose to investigate the role of fibrin deposition without altering FBG production using dabigatran, a competitive thrombin inhibitor for reducing fibrin formation (55). The severe exacerbating effects of dabigatran treatment on top of the experimental colitis symptoms shown herein indicated that fibrin is important for preventing epithelial damage when the intestine is challenged. It is noteworthy that dabigatran alone had no significant effect on any of the tested parameters. This protective role appears to fall into the more general role of FBG in hemostasis and wound repair described for several tissues and organs (81). Interestingly, FBG circulating levels have been reported to be increased in patients with active inflammatory bowel disease (82), whereas some loci including that one previously reported to be related to CD (83) were identified in a genome-wide association study to be related to plasma levels of FBG (84). There is, however, evidence that FBG is not always protective and can participate into mechanisms that contribute to disease (16, 18). For instance, in a mouse FBG knockout model, FBG deficiency appears to attenuate development of radiation enteropathy (85) and adenoma formation following a DSS/azoxymethane challenge (19), although bleeding events associated with colitis were worsened in this latter case. In mice expressing a mutant form of fibrinogen that retains clotting functions but lacks the binding site for the leucocyte receptor αMβ2, DSS/azoxymethane was successfully used to demonstrate a link between FBG and the development of inflammation-driven colonic malignancy (19). In another set of experiments, Zhang et al. (57) also showed a decrease of colonic inflammation and vascular permeability in DSS-induced colitis using the tetrapeptide GPRP known to interfere with FBG and immune cell receptors (86, 87). Taken together, these data suggest that an increase in FBG levels as observed under pathological conditions can promote mucosal inflammation through interactions with immune cells. Nevertheless, this does not exclude the protective effect of FBG as the deleterious pro-inflammatory effects mediated by FBG could be worsened if the wound healing properties of fibrin were altered as shown herein with thrombin inhibition in the DSS model.

In summary, in the intestine, fibrinogen is produced by the most mature epithelial cells and deposited in the stroma under its proteolytically processed fibrin form where it serves as a substrate for wound healing under physiological conditions such as at the site of epithelial shedding at the tip of the small intestinal villus and surface epithelium of the colon as well as under pathological conditions that require rapid epithelial repair.

## Data Availability Statement

The original contributions presented in the study are included in the article/**Supplementary Material**. Further inquiries can be directed to the corresponding author.

## Ethics Statement

The studies involving human participants were reviewed and approved by Institutional Human Research Review Board of the Université de Sherbrooke/CHUS. The patients/participants provided their written informed consent to participate in this study. The animal study was reviewed and approved by Animal Research Committee of the Faculty of Medicine and Health Sciences of the Université de Sherbrooke.

## Author Contributions

AS, GC, and JFB designed experimental studies, interpreted the data, and wrote the manuscript. AS, GC, VR-N, TK, and IT performed the experiments and acquired and analyzed the data. JFB and NP supervised and obtained funding. All authors contributed to the article and approved the submitted version.

## Funding

This work was supported by grants from the Canadian Institutes of Health Research (MOP-97836 and MOP-123415 to JFB) and the Natural Sciences and Engineering Research Council of Canada (RGPIN-2017-05489 to JFB). JFB was the recipient of the Canadian Research Chair in Intestinal Physiopathology. JFB and NP are members of the FRSQ-funded Centre de Recherche of the CHUS.

## Conflict of Interest

The authors declare that the research was conducted in the absence of any commercial or financial relationships that could be construed as a potential conflict of interest.

## Publisher’s Note

All claims expressed in this article are solely those of the authors and do not necessarily represent those of their affiliated organizations, or those of the publisher, the editors and the reviewers. Any product that may be evaluated in this article, or claim that may be made by its manufacturer, is not guaranteed or endorsed by the publisher.

## Supplementary Material

Supplementary Figure 1 Efficiency of dabigatran treatment. Efficiency of thrombin inhibition by dabigatran was evaluated by measuring blood coagulation time that increased approximately five times (n = 3, p < 0.0001).

Supplementary Figure 2
*FBG* transcript analyses in the colon of mice treated with dabigatran and DSS. Colon samples from the mice treated as above were also processed for RNA extraction and qPCR for *FBG* analyses. Data expressed as median fold relative to control with interquartile ranges. n = 6–8 per group.
