# Supplementary figures and images for: Fibrin(ogen) Is Constitutively Expressed by Differentiated Intestinal Epithelial Cells and Mediates Wound Healing
Source: Front Immunol. 2022 Jun 22;13:916187. doi: 10.3389/fimmu.2022.916187 (PMC9258339; doi:10.3389/fimmu.2022.916187)

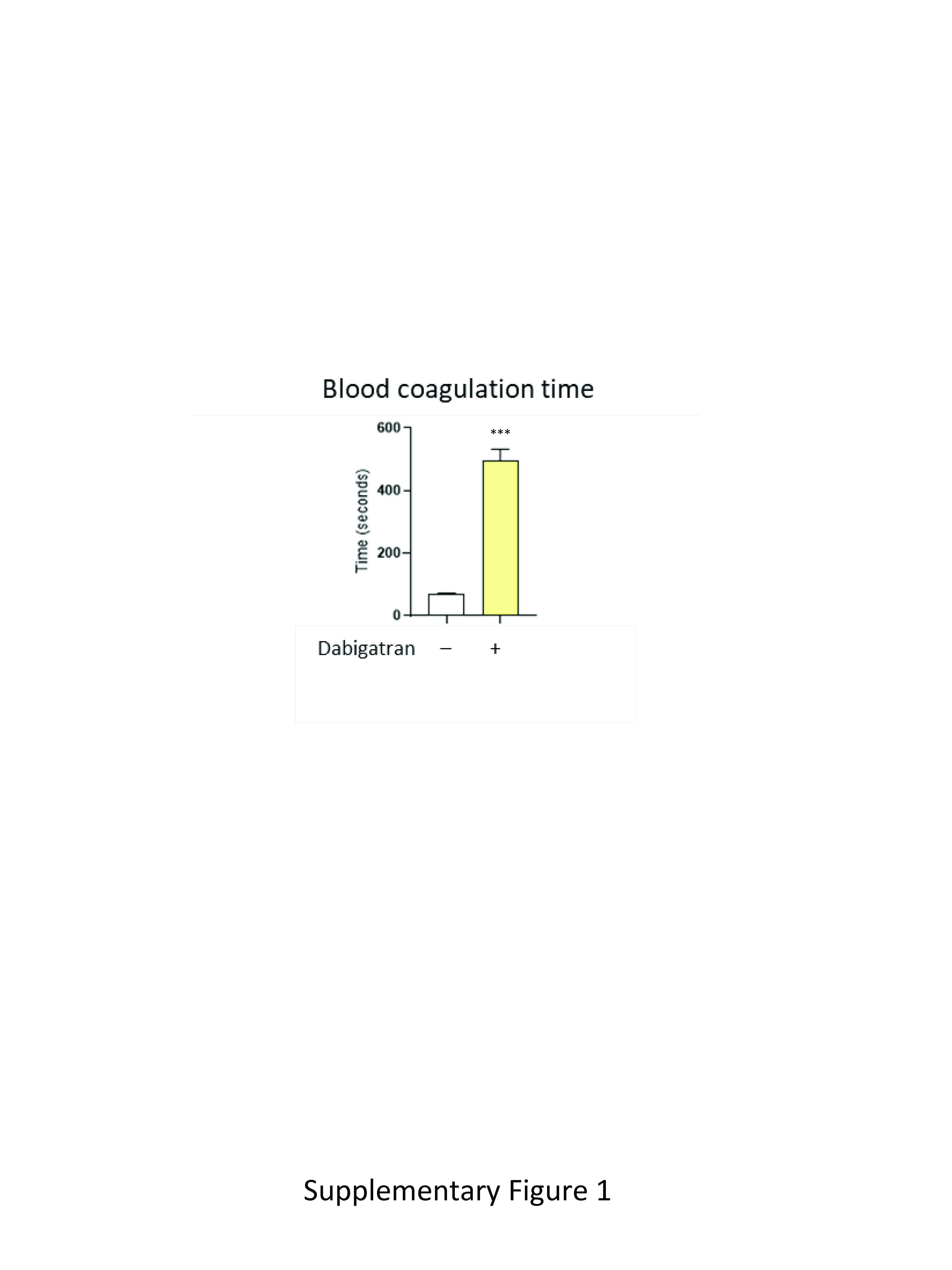

Supplement: Supplementary Figure 1 — Efficiency of dabigatran treatment. Efficiency of thrombin inhibition by dabigatran was evaluated by measuring blood coagulation time that increased approximately five times (n = 3, p < 0.0001). [file Image_1.tif]

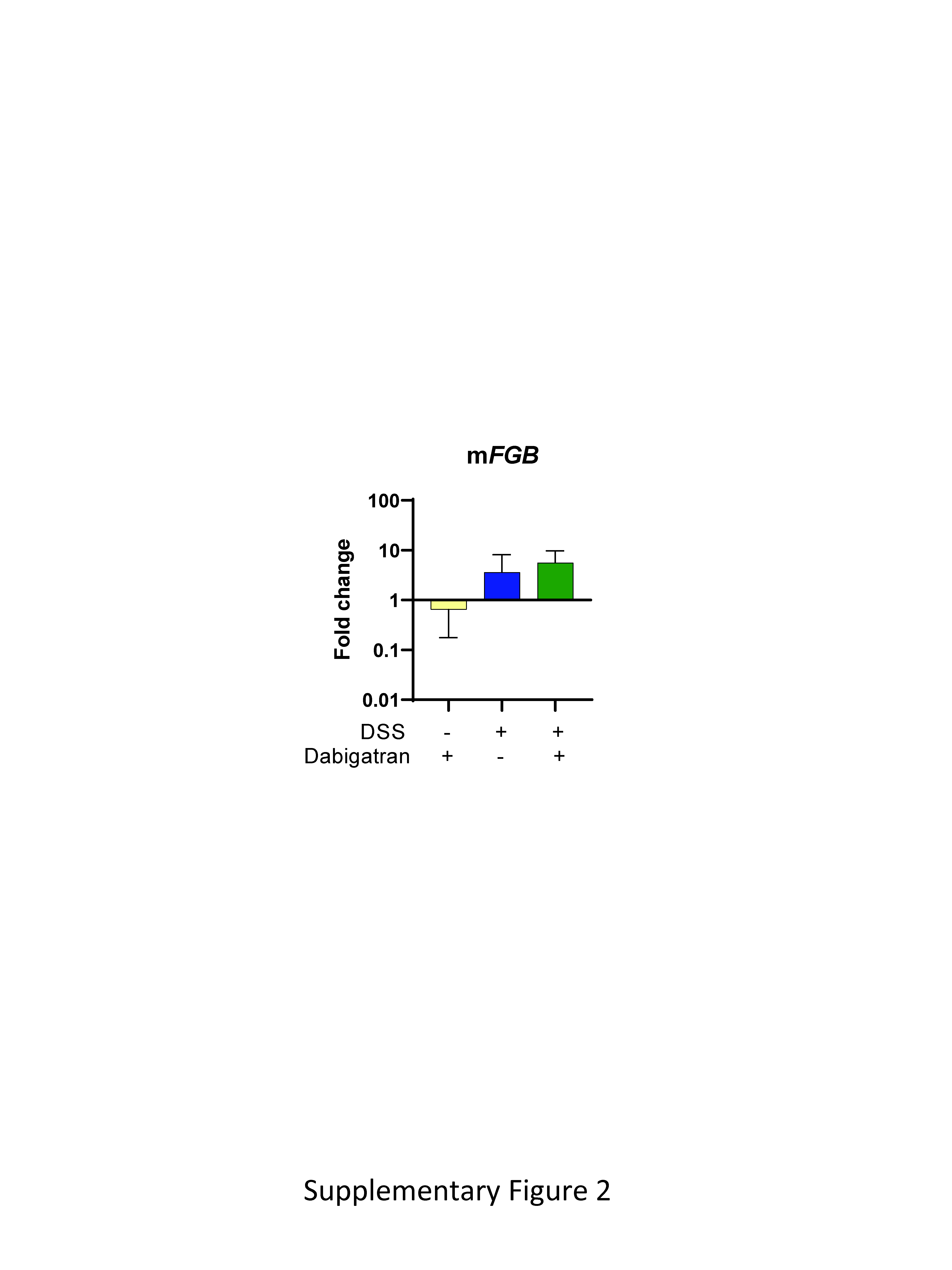

Supplement: Supplementary Figure 2 — FBG transcript analyses in the colon of mice treated with dabigatran and DSS. Colon samples from the mice treated as above were also processed for RNA extraction and qPCR for FBG analyses. Data expressed as median fold relative to control with interquartile ranges. n = 6–8 per group. [file Image_2.tif]
